# The Global Pandemic of Falsified Medicines: Laboratory and Field Innovations and Policy Perspectives

**DOI:** 10.4269/ajtmh.15-0221

**Published:** 2015-06-03

**Authors:** Gaurvika M. L. Nayyar, Joel G. Breman, James E. Herrington

**Affiliations:** Johns Hopkins Bloomberg School of Public Health and Johns Hopkins Carey Business School, Baltimore, Maryland; Fogarty International Center, National Institutes of Health, Bethesda, Maryland; Gillings Global Gateway, Gillings School of Global Public Health, University of North Carolina at Chapel Hill, Chapel Hill, North Carolina

## Introduction

This supplement to the *American Journal of Tropical Medicine and Hygiene*, entitled “The pandemic of falsified medicines: laboratory and field innovations and policy perspectives,” showcases 17 articles on detection technologies and methods, field surveillance data, multisectorial perspectives, and policy interventions and recommendations needed to create a coordinated and effective response to curb the pandemic of poor-quality medicines. The goal of this special issue is to alert scientists, public health authorities, and decision makers to the problem of poor-quality drugs and to take prompt actions to mitigate and resolve the growing peril.

**Figure 1.**
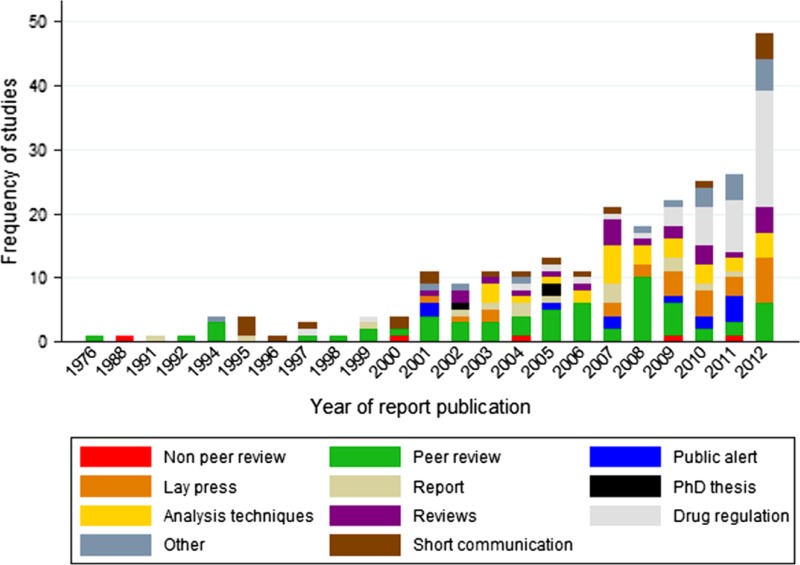
Increasing reports of poor-quality antimalarials, 1976–2012, Worldwide Antimalarial Resistance Network (WWARN), Antimalarial Quality Surveyor.^5^

Poor-quality medicines are a real and urgent threat to decades of success in global public health, particularly for programs combating human immunodeficiency virus/acquired immunodeficiency syndrome (HIV/AIDS), tuberculosis, and malaria where mortality rates have seen dramatic declines worldwide.^1^–^3^ Safe, effective, high-quality, and affordable medical products are essential to positive and equitable health outcomes for all, as noted by the U.S. Food and Drug Administration (FDA) Commissioner, Margaret Hamburg, in her Foreword to this supplement.^4^ Although previously thought to be limited to low-income countries with weak pharmaceutical regulatory systems and problems with antimalarials (Figure 1

), increasing reports of a large variety of poor-quality medicines and medicinal products, such as vitamin supplements, in high- and middle-income economies are illustrative of the pandemic nature of this problem.^5^–^7^ It is estimated that falsified medicines result in $75 billion in illegal annual revenues to criminals and have caused prolonged, severe illness and deaths worldwide; this figure needs more precision.^8^ The increasing number of countries reporting breaches of the supply chain, and products being falsified, along with the recent doubling of articles published on “fake drugs” every 5 years in PubMed indicate the problem is of pandemic proportions and growing. This may be an underestimate of the problem. Incidents regarding the distribution and use of poor-quality pharmaceuticals often go unreported due to poor surveillance systems and are kept from the public record by governments and pharmaceutical companies.^9^ Further clouding the problem is an ongoing debate and confusion over terms related to poor-quality medicines.^10^,^11^ In this summary, we use the term falsified as a synonym for counterfeit, devoid of considerations of intellectual property. We classify poor-quality drugs into three main types: falsified (intentional fraudulent manufacturing), substandard (unintentional errors caused in manufacturing), and degraded (medicines that become poor quality after manufacturing because of poor storage environments or handling).

In 2013, an estimated 122,350 deaths in children under 5 years of age in 39 sub-Saharan African countries were associated with the consumption of poor-quality antimalarials, representing 4% of all under-five deaths, as reported by Renschler and others.^12^ The impact of falsified and substandard medicines goes beyond the morbidity and mortality affecting vulnerable patients and extends to increased microbial resistance, when active drug is in low amounts in the product; existing drug-resistant microbes in patients can be spread by mosquitoes and other vectors when no active ingredient is present. In addition, socioeconomic losses and loss of public trust are associated with poor-quality medicines, all of which jeopardize years of global public health success and investments.^13^

Over the last decade, many new stakeholders have joined the cause to combat poor-quality medicines, yet little tangible progress has been made. Moreover, the problem continues to spread globally, creating an even greater challenge to cooperation among stakeholders, many with limited resources.^8^,^10^,^13^ The need is urgent for collaboration among those with expertise in policy, technology, surveillance, and logistics to secure global medicine supply chains.

## New and Promising Detection Technologies

Diagnostics are at the heart of any successful epidemic response effort; they are crucial to the ability of national regulatory agencies and the global health community to take action against the pandemic of falsified medicines by identifying them before market entry and contact with patients. Testing for poor-quality medicines is challenging because of the high cost of traditional laboratory-based analytic chemistry methods, lack of training in forensic techniques for packaging and chemical analysis, and the large sample sizes needed to conduct representative and generalizable studies in the field.^13^ Fortunately, over the last 5 years, research in detection technologies has expanded with over 42 unique detection technologies available to address poor-quality medicines, of which over half are commercially available.^14^ However, there are a lack of harmonized, agreed-upon detection standards and a scarcity of effective, affordable, rapid, and portable detection technologies that can be readily brought to scale; this is allowing poor-quality medicines to continue to contaminate national drug supply systems. This supplement includes research on four novel technologies that are promising in this field.

Weaver and Lieberman introduce a library of chemical color tests embedded on an inexpensive paper card to presumptively identify formulations corresponding to low-quality antimalarial drugs. Although this test may be relevant to low-income settings because of its portability and low-cost profile, it requires comparison to authentic colorimetric samples, many of which are not currently available from antimalarial pharmaceutical manufacturers.^15^

Green and others share a novel colorimetric assay for the simultaneous assessment of both lumefantrine and artemether in Coartem™ tablets, manufactured by Novatis (Basel, Switzerland). They take a three-tiered approach to test for falsified medicines, including image analysis, and integrating the technology with two other novel, low-cost, fluorescence scanning devices. This very promising simple combination intervention for field settings requires caustic acids for the assay, which can be difficult to manage in remote, low-income settings.^16^

Another novel assay technology in early-stage development, described by Ho and others,^17^ consists of a detection reagent (probe) and a micro-fluidic platform to test for active pharmaceutical ingredients (APIs) of antimalarial drugs with the potential for integration into a fully automated field-ready system. Kaur and others have used chemical and bioassay techniques to test the quality of the antileishmanial drug miltefosine, a drug that has played a role in the elimination program for visceral leishmaniasis (kala-azar) in India, Nepal, and Bangladesh.^18^,^19^ Overall, these four new and promising proof-of-concept technologies face similar challenges—an absence of needed funding for field setting validation and scalability. Importantly, there is a necessity for evidenced-based policy guidance on the role of these new tools in field surveillance.

## Field Reports of Poor-Quality Medicines

Representative and generalizable epidemiological surveys on poor-quality medicines are scarce. For example, no reliable global estimates are available describing the prevalence of poor-quality medicines, in large part due to the lack of consensus on harmonized international definitions of poor-quality medicines and surveillance methods.^20^ World Health Organization (WHO) has attempted to develop a consensus on definitions, but there is yet no globally recognized, actionable resolution to date. No globally harmonized standards or statistically representative sampling schemes and testing protocols exist for surveillance to inform a regulatory and legal response against those who knowingly and deliberately distribute falsified and substandard medicines.^7^,^9^,^10^ Adding to this complexity, national regulatory authorities are inadequately trained, equipped, and funded to conduct routine and systematic surveillance of their drug supply systems. Limited funding from donors to provide support for regulatory systems strengthening and medicines quality monitoring affects the capacity of progressive governments and engaged civil society organizations to collect statistically representative samples of medicines to test for product quality.^21^ Ultimately, this creates a vicious cycle of a poor evidence base perpetuating the lack of political will and global accountability in responding to this scourge. This supplement offers insight into this issue from seven high-quality field surveys, each using unique and robust sampling methodologies and testing protocols, thus offering an important snapshot of recent field data on the quality of lifesaving medicines in low- and middle-income economies.

Collectively, across the seven quality survey studies in the supplement, ~16,800 samples of antimalarials, antituberculosis medicines, antibiotics, and antileishmaniasis medicines were tested for quality and an estimated 9–41% of specimens failed quality specifications.^22^–^28^ These studies used unique sampling and data collection strategies, including samples drawn from nationally representative surveys, government and Interpol seizures, “mystery shoppers” (unknown to the vendor), convenience samples, and overt and repeat randomized surveys. For instance, Yeung and others conducted sampling using mystery shoppers and overt surveys in the epicenter of antimalarial drug resistance in Cambodia. Survey results showed that, of 291 samples tested, mystery clients were more likely to receive an oral monotherapy, which is banned in the country. Results also found that over 30% of the medicines collected did not fall in the 85–115% range of the stated API and that there were no falsified antimalarials; most of these failures (58, 19.9%) were in the 75–84% unacceptably low range.^22^ Overall, the findings of this study reflect the positive impact of the country’s effort to ban monotherapies and to control drug quality. Also noted at the country level, Lalani and others randomly sampled 134 antimalarials from 60 outlets (public and private) in Afghanistan and found 26% failed disintegration testing, as outlined in the Global Pharma Health Fund-MiniLab^®^ (GPHF, Frankfurt, Germany), and a subsample of sulfadoxine/pyrimethamine and quinine compounds failed U.S. Pharmacopeial (USP) tolerance limits (32.4%, 12/37) when assessed by in vitro dissolution testing. This study suggests that substandard drugs need to be considered within the context of poor bioavailbility, as well as insufficient API, and highlights a need for a regular and systematic medicines quality surveillance program in Afghanistan.^23^ The Artemisinin-based Combination Therapy (ACT) Consortium Drug Quality Project Team/IMPACT2 study tested the quality of artemisinin-based antimalarials from a nationally representative sample of Tanzania’s private sector. They found that while none of the 1,737 antimalarials were falsified, a minority were of poor quality; medicines lacking WHO prequalification status were more likely to be poor quality.^24^ Of the seven quality studies, results from the Medicines Quality Database at the USP Convention contributed the largest sample of medicines; 15,063 samples were collected from 17 countries of Africa, Asia, and South America. The highest proportion of failure was among antimalarials, 6.5% between 2003 and 2013 (478/7,333).^25^ Tabernero and others used a random sampling design and conducted a follow-up survey to detect poor-quality medicines in Lao People’s Democratic Republic, assessing changes over time from 2003 to 2012. Although an overall reduction in the number of poor-quality medicines was observed, the study detected that 25% (9/37) of samples were outside pharmacopeial limits.^26^

Fadeyi and others tested 35 samples of antibiotics purchased in Ghana, Nigeria, and the United Kingdom that were manufactured in six countries (China, Ghana, India, Nigeria, Ireland, and the United Kingdom) using GPHF MiniLab^®^ thin layer chromatography (TLC), in vitro dissolution, and high-performance liquid chromatography photodiode array detection (HPLC-PAD). All samples of amoxicillin released the expected amount of API within time and met the USP tolerance limits. Of the 15 co-trimoxazole samples purchased, six (40.0%) (two from Ghana and four from Nigeria) met USP tolerance limits but nine (60%; three from Ghana and six from Nigeria) did not. Test results using MiniLab^®^ TLC were inconsistent, highlighting the need to invest in techniques such as HPLC and dissolution testing.^27^ On the other hand, Yong and others obtained samples of antibiotics and antimalarials from seizures conducted by Interpol and medicine regulatory authorities and observed that one-third of all antibiotic and antimalarial samples had API compositions outside pharmacopeial specifications (< 85% or > 115% API). This study offers a model example of how multiple national and international enforcement and regulatory agencies have come together to respond to poor-quality medicines.^28^

The majority of the publicly accessible rapid alert, reporting, and response networks and databases for detecting, storing, and sharing information on global breaches in supply chain security are voluntary, including among others the WHO Rapid Alert System, Medicines Quality Database, WHO Western Pacific Region Rapid Alert System, and Worldwide Antimalarial Resistance Network (WWARN) AQ Surveyor.^21^,^29^,^30^ However, while reporting is improving, few countries submit their full complement of notable drug-quality events reports to these databases due in part to not understanding the benefits of such reporting. This prevents decision makers and the public from taking action, because data are incomplete, nonrepresentative, fragmented, and/or confidential. This complicates the challenge of gathering and galvanizing the necessary evidence for technical assistance and regulatory action.^9^ Fortunately, Mackey and others report on novel results from the Pharmaceutical Security Institute’s Counterfeit Incident System (CIS), a nonpublic database collecting information on incidents of diversion, theft, and fraud from 28 pharmaceutical companies. Of the 1,510 identified CIS reports involving falsified medicines, 28% reported China as the country of origin of the incident/detection. In line with other trends, the most prevalent falsified medicines were anti-infectives, mostly from Asian and Latin American regions (reported geographically) and from middle-income markets (reported economically).^31^

## Policy Perspectives

The pandemic of poor-quality medicines requires an urgent and coordinated international response. The authors in this section of the supplement argue that this can be achieved via a multisectorial response, including a global convention^32^ and through tailored national model laws.^33^ For example, Attaran argues that poor-quality medicines, as a criminal enterprise, exists because present laws are unbalanced: “… the free trade laws that cause medicines to be globally traded are not matched by criminal laws to prosecute those who illicitly traffic medicines, seize their assets, and better secure the medicine supply chain.” Trade that is both open and free yields benefits to many who would not otherwise have access to lifesaving drugs. When open and free trade fails to have adequate legal oversight, the end user can be harmed, not infrequently resulting in death, because criminality in the production and distribution of falsified and substandard medicines is left unchecked.^8^ Attaran suggests that to protect public health and secure the national drug supply chain require law reform that both stigmatizes the crimes as evil and punishes the criminals in proportion to the harm they cause. Even in developed economies, the penalties for producing and distributing falsified and substandard medicines are lamentably weak. Until recently, Canada imposed a maximum penalty of 3 years’ imprisonment and a $5,000 fine for adulterating a medicine, France levels only 3 years’ imprisonment and a €75,000 fine, while in Norway incarceration is just 4 months for this crime. In the Netherlands, the crime for production of a substandard medicine requires that the perpetrator commit the act twice in 2 years—the first violation is excused—and then the prison term is only 6 months maximum. Attaran argues that countries should ideally “have laws that target and suppress the harmful elements of the global medicine trade—the substandard and falsified medicines—without interfering with the legitimate trade in either branded or generic medicines. This is a goal that the public health community, the law enforcement community, and the pharmaceutical industry should all be able to agree on.” To this end, a *Model Law on Medicine Crime* has been drafted that stresses compatibility with a country’s existing laws and is available free online at http://papers.ssrn.com/sol3/papers.cfm?abstract_id=2530087.

Similarly, Nayyar and others suggest that to have a lasting and sustained impact, this response must focus on increasing the information on, and availability of, standardized detection technologies, national legislation bolstered by an international law/convention addressing technical–financial–legal dimensions, and, most urgently, a leading organization, with technical expertise and influence similar to that of the FDA, that can coordinate cross-sectorial stakeholders and mobilize resources in a transparent manner. These recommendations have notable and effective precedents for using international law in this way. For example, a 1929 international treaty criminalized counterfeit currency and the Framework Convention on Tobacco Control (FCTC), and its associated protocols, prohibited illicit trafficking of tobacco products. Indeed, the FCTC has realized over $250 million in new funding for global tobacco control efforts and reduced the availability of health-harming tobacco products, thus demonstrating that an international treaty can both raise needed operational revenues and have a health impact. The FCTC provides an excellent model for combatting falsified and substandard medicines.^32^,^34^

Finally, Cinnamond and Woods,^35^ of the Global Fund to Fight HIV/AIDS, Tuberculosis and Malaria (GFATM), describe the newly established Joint Inter-Agency Task Force and Global Steering Committee for the Quality Assurance of Health Products. These two proactive units, based within a major supplier of antiretroviral, antituberculosis, and antimalarial drugs, offer a systematic and coordinated approach to promoting and protecting access to safe and effective medicines in low- and middle-income countries that are recipients of the GFATM financing mechanism.

## Conclusions

The threat of poor-quality medicines presents a real and present danger to decades of effort and success by many governments, multilateral organizations, philanthropies, and private sector groups in fighting HIV/AIDS, tuberculosis, malaria, and many other conditions that have witnessed steady and significant declines in mortality worldwide. These public health triumphs are due in part to access by families to safe, effective, high-quality, and affordable medicines and medicinal products, as articulated by the FDA Commissioner. However, as shown in this supplement, survey data from over 17 countries reveal that poor-quality medicines and medicinal products represent a pandemic of grave concern to the health and well-being of populations globally, but especially those living in low- and middle-income nations where national drug regulatory systems and policies are weak or ineffective for lack of enforcement. Fortunately, new techniques for sampling and detecting falsified and substandard medicines proposed in this supplement merit further study for validity in field settings and, if proven effective, should offer venture capitalists and other funders opportunities for investment to bring these tools to scale so governments can secure their medicine supply systems. Nonetheless, no tool or technique is of any value if not backed by good governance and the rule of law. To this end, a global convention that addresses the technical–financial–legal dimensions of the pandemic of falsified and substandard medicines, coupled with a *Model Law on Medicine Crime* offers national governments valuable tools to address these weaknesses through normative guidance, evidence-based policies, and tough legal and financial penalties for those who manufacture and/or distribute falsified and substandard medicines and medical products. We hope this clarion call to action will be heard and acted upon by policy makers and leaders at international and national levels.

**Figure 2.**
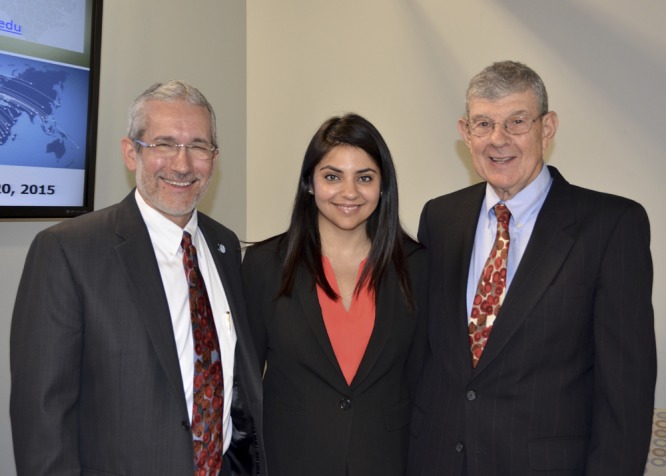
Photo of co-editors. (L–R) James E. Herrington, Gaurvika M. L. Nayyar, and Joel G. Breman.
